# How do applicants, students and physicians think about the feminisation of medicine? - a questionnaire-survey

**DOI:** 10.1186/s12909-020-1959-2

**Published:** 2020-02-11

**Authors:** D. Laurence, Y. Görlich, A. Simmenroth

**Affiliations:** 1 Department of General Practice and Study Deanery, University Medical Center Göttingen, Göttingen, 37073 Germany; 2 Department of General Practice, University Hospital Würzburg, Josef-Schneider-Str. 2/ D7, 97080 Würzburg, Germany

**Keywords:** Gender, Feminisation, Medical student, Doctor, Medical education, Attitude, Career choice

## Abstract

**Background:**

The implications of the feminisation of medicine, which is characterised by a growing proportion of female doctors, is a topic currently being debated worldwide. To date, however, there has been no systematic survey of the viewpoint of present and future doctors on this subject. The aim of the present study is to determine how future and present doctors view this trend in terms of its relevance to the medical profession and its present impacts.

**Methods:**

Of a total sample of 3813 people, 181 applicants for the winter term 2014, 590 medical students and 225 doctors of the UMG participated in this cross-sectional electronic questionnaire. The answers were analysed by means of the statistics program IBM SPSS Statistics 22. Open answers were qualitatively evaluated and categorised using the “Basiswissengeleitete offene Kategorienfindung” (Werner Früh) and coded for statistical analysis.

**Results:**

The majority of our participants favoured a balanced gender-ratio among doctors: 77% of applicants, 68% of students and 61% of doctors rated this as important or very important. The results from the student and applicant groups differed concerning female gender. When answering in the role of a patient, the doctor’s gender was found to be more relevant than when the participants were answering in the role of the doctor. The majority of the respondents opined that feminisation had had an impact on their workplace: particular factors included part-time work, work-related organisation and the diversity of the medical profession. Commentaries were mostly categorised as negative.

**Conclusions:**

The feminisation of medicine was viewed largely critically by the participants of this study. The respondents evaluated gender as being relevant for the medical profession and favoured a diverse workforce; however, the significance of one’s own gender in medical practice was underrated in comparison, implying a need for more awareness of the effect of a doctor’s gender on the patient-doctor-relationship. The mainly negative comments concerning the impact of feminisiation on work organisation, work satisfaction and patient care show the need for further research and action to adapt current medical work practices to the changing demographics in order to improve work satisfaction and quality of care.

## Introduction

Like many professions, medicine is undergoing continual changes that reflect transitions within the society which it serves. The qualities perceived as necessary to become a doctor and the definition of a ‘good doctor’ have been the subject of repeated discussions. For example, in the past, a paternalistic doctor-patient relationship held sway within the medical profession; however, this has been gradually supplanted by a caring partnership with the patient [1]. ‘New’ qualities such as empathy, good communication and social skills are now both desired and expected.

One of the relevant changes the medical profession is currently facing is the increasing fraction of women among doctors, the so-called ‘feminisation of medicine’. In Germany, the percentage of female doctors has increased from 34% in 1991 to 46% in 2014 [2]. Considering that, since 2004, more than 60% of Germany’s medical students have been female, further increases in this percentage can be expected in the future [3]. This trend is not exclusive to Germany, but rather constitutes a worldwide phenomenon. In many ways this is a positive development: for example, a majority of female patients are better able to relate to a physician of the same sex [4, 5] and this may lead to beneficial outcomes of their care. Also, female doctors generally exhibit higher levels of qualities such as empathy that are currently desired in the medical profession [6]. Furthermore, this trend can be viewed as a success for female emancipation after a long history of limited access for women to higher education and work. However, this trend also brings potentially problematic consequences. In the “OECD Health Working Papers 2006” (Organisation for Economic Co-operation and Development), the increasing proportion of female doctors has been cited as a major contributing factor to the insufficient physician supply that is of growing concern in many OECD member states [7]. This study states that the time worked by female doctors is, on average, up to 15 h less per week than that of male doctors; additionally, many women do not reenter the profession after family leave. The feminisation of medicine has also been cited as one of the contributing factors to workforce shortages in specific fields such as medical research [8], and surgery [9], which have been observed as less attractive to women [8]. Moreover, medical leadership positions are still mainly occupied by men: Rochon et al. [10] partially attribute this to the difficulties women face in combining family life with a medical career, although a lack of identification with the profession and vague career goals might also be contributing to the underrepresentation of women in higher positions [11]. An additional factor may be cultural biases that lead to women being perceived as less successful and thus hinder them from being selected for positions of leadership [12].

In the relevant literature, the causes of the feminisation trend have been the subject of much controversy. On the one hand, Klemperer [1] argues that the recent loss of prestige and autonomy associated with the medical profession makes it less attractive to men. On the other hand, studies have shown that males are outperformed by females in both secondary school and tertiary education worldwide [13], decreasing their probability of being admitted to prestigious programs such as medicine. Budde [14] points to discrimination in school as a potential source of this “gender gap”, whereby classic “boy behavior” often is censured and classic “girl behavior” rewarded. This not only appears to discourage boys from believing in their own potential from a young age [15], but also negatively influences teachers’ grading of them [16, 17]. This effect may be partially balanced by the negative influence of gender stereotypes experienced by girls with regard to STEM subjects (science, technology, engineering, mathematics), which in many countries are essential for admission to medical school. Girls’ lower interest and performance in STEM subjects might be related to the reduced expectations parents in many OECD countries have towards girls pursuing a career in a STEM field [13], as well as societal perceptions of STEM fields as “masculine” [18]. Studies have also suggested a gender bias to exist in teachers’ grading of STEM subjects [19, 20].

The effects of the gender imbalance on the medical profession are manifold: the literature shows various aspects of doctor-patient interactions in which the gender of the doctor is perceived to be significant. Aside from patients’ preferences for a specific gender, which can be gender-concordant or discordant [4, 21], a generally positive effect of “gender-matching” in the doctor-patient relationship [22] and treatment in specific settings, e.g. psychotherapy [23, 24], has been observed. Additionally, reviews have shown male and female doctors to exhibit different characteristic strengths and weaknesses, all of which are important for the medical profession in different settings: for example, women typically are more empathetic and have superior communication skills [6], whereas male doctors show greater confidence in broaching sensitive issues such as patients’ sexual history [25] and are, in general, more work-efficient [26].

Moreover, Boylan & Grant [27] note how important it is that members of the medical profession reflect the society they serve – in ethnicity, race and gender – and thus conclude that the composition of members of the medical profession is, in itself, of importance to society. Therefore, with regard to both the importance of diversity within the profession and the relevance of gender for the doctor-patient relationship in various contexts, the present continuous increase of female doctors deserves attention.

Although this topic has been discussed at length in the literature, to our knowledge, members of the medical profession themselves have not yet been interrogated on their viewpoints and experiences as pertaining to this matter. The aim of this study is to contribute to the analysis and understanding of the ‘feminisation of medicine’ through a survey of individuals within the profession, to gauge present and future implications of this phenomenon, and to provide suggestions for future research.

## Methods

For this cross-sectional study, we conducted a partly standardized electronic survey of individuals in the medical profession at the University of Göttingen in Germany.

### Participants

We addressed medical school applicants, current medical students, and doctors of the UMG as potential future and current representatives of the medical profession. German medical-school education takes 6 years, of which the first two are preclinical, years 3 to 5 are clinical, and year six consists of clinical rotations. Of a total possible sample size of 3813 people, 181 applicants for the winter term 2014, 590 medical students and 225 doctors of the UMG participated in this survey. Our survey was included in an evaluation questionnaire reviewing the admission process that UMG applicants filled out by default in August 2014. Students and doctors (including residents, consultants, principal consultants and professors) were sent an email-link for the online survey as well as a PDF copy of the survey via their respective electronic mailing lists in November–December 2014.

### Measures and procedures

For the generation of the survey items, we conducted open interviews with four persons at different stages of their medical careers: a medical school applicant, a medical student, a resident and a principal consultant. From these interviews, we derived the following relevant topics associated with the “feminisation of medicine”: reasons for the trend (including the current selection process of medical students in Germany), current and future implications of the feminisation, and the relevance of these implications to the medical profession. From these topics, we constructed the applicants’ survey; this also doubled as a pilot study, since the main focus of our survey was on students and doctors. Following receipt and initial analysis of the applicants’ responses, two out of six survey items were partially modified for the doctor and student surveys through categorization of the analysed free-text responses and conversion into closed multiple-choice questions with the option of a free-text answer, as the free text responses to these two items were found to be repetitive. Only one of these items is discussed in this paper: “When you are a patient, is the gender of your doctor important to your bond of trust to him/her? *If yes, explain…*”. The item “Based on your experience, how important is the gender of a doctor (in your specialty) for the bond of trust to their patients?” was added to the students’ and doctors’ survey as it only applied to these survey subgroups. The item “Does the trend of the feminisation of medicine have implications on your specialty field?” was generated from the interviews and added to the doctors’ survey as it was only relevant to this survey subgroup.

Reasons for the feminisation trend will be addressed in a future publication; here we focus on its implications and their relevance to the medical profession.

### Statistical analysis

The answers were analysed by means of the statistics software IBM SPSS Statistics 22; the tools used were descriptive statistics (e.g. mean, cross tabulation), the Mann-Whitney-U-test and Pearson’s chi-squared test. Open answers were qualitatively evaluated and categorized using the “Basiswissengeleitete offene Kategorienfindung” (BoK, technique of categorization led through basic knowledge) [28] and coded for statistical analysis. The BoK follows a three-step text analysis. First, relevant passages for the research topic are extracted, condensed, segmented by propositions and categorized. In the second step, attributes are selected from within the extracted propositions, and are semantically analysed and labelled. Finally, these labelled text passages are grouped into final categories, which can be coded and used for further analysis. Unanswered items were excluded from the analysis, though other completed items by the same participant were processed.

### Privacy protection and ethics approval of research

The study was conducted anonymously; the participants’ generated data were assigned to automatically generated case numbers. An approval of our study by the ethics committee of the UMG was obtained in October 2014.

## Results

### Sample composition and response rate

The response rate among the applicants, medical students and doctors was 100, 25 and 17% respectively. Among the medical students, the participation rate was highest for clinical students (33%), followed by students in clinical rotation (26%) and preclinics (22%). In the group of doctors, the participation rate was lowest for residents (16%) and higher for fellows (31%), attending physicians (24%) and heads of department (31%). Doctors working in medical research were also included in the survey and partly make up the doctor subgroup “UQ”.

In relation to the survey population, our sample of students is representative of the total student body of the UMG in terms of gender ratio and number of students per stage of medical education. Overall, a larger fraction of female doctors participated in our survey relative to the fraction of female doctors in the population. Also, proportionally more consultants and principal consultants answered our questionnaire than residents in comparison to the overall survey population (Table 1).

**Table 1:** Composition of sample

Survey group	Subgroup	Male	Female	*UQ*^b^	Total
		N (%)^a^	N (%)	*N*	N (%)
Applicants		66 (36.5%)	115 (63.5%)	*0*	181 (100%)
	*Total*^c^	*66 (36.5%)*	*115 (63.5%)*		*181 (100%)*
Medical students	Preclinical^d^	71 (30.7%)	160 (69.3%)		231 (100%)
	*Total*	*402 (39.1%)*	*626 (60.9%)*		*1028 (100%)*
	Clinical^e^	92 (35.2%)	169 (64.8%)		261 (100%)
	*Total*	*499 (38,6%)*	*794 (61,4%)*		*1293 (100%)*
	Clinical rotation^f^	23 (31.1%)	51 (68.9%)		74 (100%)
	*Total*	*?*	*?*		*281 (100%)*
	UQ				24
	Subgroup total	187 (32.9%)	381 (67.1%)	*2222*	590 (100%)
	*Total*	*901 (38,8%)*	*1420 (61,2%)*		*2321 (100%)*
Doctors	Residents	36 (44.4%)	45 (55.6%)		81 (100%)
	*Total*	*262 (52,4%)*	*238 (47,6%)*		*500 (100%)*
	Fellows	14 (37.8%)	23 (62.2%)		37 (100%)
	*Total*	*61 (51,3%)*	*58 (48,7%)*		*119 (100%)*
	Att. Physicians^g^	34 (68.0%)	16 (32.0%)		50 (100%)
	*Total*	*155 (73,8%)*	*55 (26,2%)*		*210 (100%)*
	Heads of dep^h^	12 (80.0%)	3 (20.0%)		15 (100%)
	*Total*	*44 (91,7%)*	*4 (8,3%)*		*48 (100,0%)*
	UQ^i^				42
	Subgroup total	112 (52.1%)	103 (47.9%)	10	225 (100.0%)

^a^*N* = 100.0% refers to the total number of survey participants, who answered this question

^b^*UQ* unanswered question. This question was not answered by participant

^c^Total refers to the total population of the respective subgroups from which the sample was drawn

^d^Preclinical years comprise of the first 2 years of German medical school education

^e^Clinical years comprise the years 3 to 5 of German medical school education

^f^Clinical rotations make up year 6 of German medical school education

^g^Attending physicians

^h^Heads of department

^i^Dentists and doctors working in medical research were also part of the email list. From an item not included in this paper we know that 34 medical researchers and 8 dentists participated in the study which we assume make up the number of UQ in this sample

#### Importance of an evenly balanced gender ratio

The item “How important is it to you that both genders are represented in equally strong numbers among doctors?” was included in all three groups’ surveys. Participants could choose their answer from a 5 point answer scale, which ranked from “very important” to “very unimportant”. Here, a clearly positive verdict was reached: 77% of applicants, 68% of students and 61% of doctors rated a balanced gender-ratio among doctors as important or very important (Table 2).

**Table 2:** Importance of an evenly balanced gender ratio

Survey group	Item answer	Male	Female	Total
		N (%)^a^	N (%)^a^	N (%)
Applicants	Very important	23 (35.9%)	56 (50.0%)	79 (44.9%)
	Important	22 (34.4%)	34 (30.4%)	56 (31.8%)
	Partly important	9 (14.1%)	15 (13.4%)	24 (13.6%)
	Unimportant	6 (9.4%)	5 (4.5%)	11 (6.3%)
	Very unimportant	4 (6.3%)	2 (1.8%)	6 (3.4%)
	*UQ*			*5*
	Total	64 (100.0%)	112 (100.0%)	176 (100.0%)
Medical students	Very important	42 (22.5%)	103 (27.0%)	146^b^ (25.6%)
	Important	74 (39.6%)	167 (43.8%)	243^c^ (42.6%)
	Partly important	38 (20.3%)	76 (19.9%)	114 (20.0%)
	Unimportant	24 (12.8%)	26 (6.8%)	50 (8.8%)
	Very unimportant	9 (4.8%)	9 (2.4%)	18 (3.2%)
	*UQ*			*19*
	Total	187 (100.0%)	381 (100.0%)	571 (100.0%)
Doctors	Very important	27 (24.1%)	25 (24.3%)	53^a^ (24.5%)
	Important	42 (37.5%)	36 (35.0%)	78 (36.1%)
	Partly important	24 (21.4%)	22 (21.4%)	46 (21.3%)
	Unimportant	15 (13.4%)	18 (17.5%)	33 (15.3%)
	Very unimportant	4 (3.6%)	2 (1.9%)	6 (2.8%)
	*UQ*			*9*
	Total	112 (100.0%)	103 (100.0%)	216 (100.0%)

^a^The percentage refers to the respective ratio of female or male survey participants

^b^One entry for “very important” without declaration of gender

^c^ Two entries for “important” without declaration of gender

The Mann-Whitney-U-Test was utilized to determine differences in the participants’ response pattern based on gender. Results showed significant differences within both the student and applicant groups: female participants found a balanced gender ratio more important than males (*p*-value for applicants and students was 0.038 and 0.02 respectively). However, no significant difference was found in response pattern between male and female doctors.

#### Importance of gender for doctor-patient relationship

To determine the importance of gender for the doctor-patient relationship, the following item was generated: “Based on your experience, how important is the gender of a doctor (in your specialty) for the bond of trust to their patients?” This item was included in the student and doctor surveys. In the following item, participants were given the opportunity to explain their standpoint in a free-text answer.

There was no clear answering tendency in the student group (see Table 3). Pearson’s chi-square tests showed no significant differences in answering pattern based on the gender of the students or their year in medical school.

**Table 3:** Importance of gender for doctor-patient relationship

Medical Students: *Based on your experience, how important is the doctor’s gender for the bond of trust to their patients?*		Doctors: *Based on your experience, how important is the gender of a doctor in your specialty for the bond of trust to their patients?*	
Response Category	N (%)	Response Category	N (%)
Very unimportant	37 (6.6%)	Very unimportant	29 (14.4%)
Unimportant	274 (49%)	Unimportant	108 (53.7%)
Important	228 (40.8%)	Important	60 (29.9%)
Very important	20 (3.6%)	Very important	4 (2.0%)
Total	559 (100.0%)	Total	201 (100.0%)
*UQ*	*31*	*UQ*	*24*

When asked to explain their position, most students who rated gender as “important” or “very important” stated that the doctor’s gender mattered in intimate body examinations. Others commented that patients generally preferred a doctor of the same or a specific gender. Age and the individual history of the patient were also mentioned as factors: students observed that these might lead to problems opening up to the opposite sex. Students also frequently observed discrimination towards female doctors from male colleagues or elderly patients. According to the comments, female doctors were seen as less competent as male doctors because of their gender.

For students who rated gender as “unimportant” or “very unimportant”, the following factors rather than gender were named as being crucial to win the patient’s trust: competence, character, empathy, social/communication skills, and a confident appearance (in descending order of frequency). Students still noted, however, that a doctor’s gender plays a role when it comes to intimate body examinations.

Doctors overall rated gender as “unimportant” for the doctor-patient relationship (Table 3). No significant differences were found based on the gender of the doctor (Pearson’s chi-squared test).

In the free-text answers, doctors who rated gender as “important” or “very important” stated most commonly that patients tend to open up more to a doctor of the same gender. The second most common response was the lack of recognition given to female doctors in comparison to male doctors. Other responses named elderly patients as more resistant to accepting female doctors. Gender was also seen as important in the field of psychotherapy.

Similar to student responses, doctors who found gender “unimportant” declared other factors such as competence, empathy, character, and communication skills as crucial for the doctor-patient relationship. Doctors who rated gender as “very unimportant” most frequently mentioned that there was hardly any patient contact in their field as an explanation for their answer.

Doctors and students were also asked how they rated the importance of the doctor’s gender when they were in the position of being a patient themselves: “When you are a patient, is the gender of your doctor important to your bond of trust to him/her?” It is noteworthy that doctor’s gender was found to be more relevant in this scenario, with 43% of doctors and 52% of the students affirming the gender to be important.

#### Implications of feminisation

We questioned doctors on the implications of feminisation on their workplace: “Does the trend of the feminisation of medicine have implications on your specialty field?” Doctors were asked to answer with “yes” or “no” and explain their choice in the subsequent open question.

A majority of 57% answered in the affirmative. Table 4 shows topics and categories of the most common free-text answers (the minimum to be included here was set to 5 for each category) as well as sample responses for each category. Doctors noted an increase of female doctors, part-time work, work absences and remarked on resulting struggles to organize overnight and weekend shifts. A loss of diversity in the medical profession was commented upon, as were demands from doctors for working-time models and staffing levels to be adapted to the new work preferences and absences. Other respondents remarked on a decline of passion for a career in medicine and for the profession itself, as well as a lack of women in higher ranks. Some doctors reported a negative working climate caused by an increased workload for male and childless doctors. Lastly, comments showed that preferences for a male therapist or doctor could not be met despite some patients’ wishes.

**Table 4:** Implication of feminisation on the workplace

Topics	*N*^a^	Sample of responses
1. Doctor team composition		
Relative increase in part-time workers.	31	*“More and more part-time work. There is a decreasing willingness to take over on-call duties because of family reasons.”*
Increase of female doctors.	29	*“Almost only female colleagues! (except for the attending doctors)”*
		*“A lot of female applicants for open positions, only few male applicants.”*
2. Organisation of hospital work		
Increase of work absences due to pregnancy and maternity/parental leave.	28	*“High ratio of female doctors in our department, resulting in work absences because of maternity leave, pregnancy, sickness of children.”*
Increased difficulties in organising overnight/weekend shifts due to part-time work or work absences.	16	*“The organisation of overnight/weekend shifts with an increased ratio of mothers demands more flexibility from all the doctors. In my opinion, also more staff are needed. Gaps in the staffing level during pregnancy, maternity leave and parental time cannot simply be compensated for by substitute staff. Instead, incentives should be created.”*
3. Diversity of the medical profession		
Profession becomes lopsided because of missing male colleagues.	14	*“Women are highly overrepresented.”*
		*“There are way too few male therapists who can be healthy “role models” especially to male patients with pathological developments.”*
		*“Our wards are almost entirely made up of female therapists and doctors, which is unfavourable for some of our patients. There is a high demand for part-time work which makes teamwork and additional scientific research difficult.”*
Less time for invasive therapy.	5	*“There is a tendency for less invasive therapy because of an increased ratio of female doctors.”*
4. Demand for structural changes		
Adjustments of working-time models and staffing levels are necessary to adapt to the feminised workforce.	17	*“The present working-time models only allow a limited use of mothers. This results in a surplus work load for male doctors as they have to cover for the strenuous overnight/weekend shifts.”*
		*“There are problems in organising overnight/weekend shifts: part-time working mothers, but a lack of job-sharing work models and adjustments for part-time work which are highly needed! Because: feminisation of medicine!!!”*
		*“More women, more mothers, more part-time work, more pregnancies = less workforce for overnight shifts* etc.*, leading to a build-up of work. Part-time work has been accepted by our institution but the concept has not been well translated into our daily hospital work.”*
		*“Without making a judgement here, it can be observed that a fluctuation of our staffing levels because of pregnancy and sickness of children at home is more frequent with female colleagues. If the institute was better equipped to compensate for these fluctuations, it wouldn’t be a problem. But it is not.”*
5. Working morale		
Family-friendliness of the profession has become more important than career; less passion/motivation for the profession.	13	*“Almost all attendees are male. Women oftentimes leave the clinic.”*
		*“Less women want to be self-employed; being an employee is more advantageous for family planning. There is a tendency towards shared practices.”*
		*“As there are no overnight or weekend shifts in pathology, we have an increasing ratio of female doctors. Unfortunately, it seems that many female doctors make that choice because of the working time instead of a passion for the field. This results in a decrease in motivation and zeal and therefore in work quality. The actual important function of a pathologist is not appreciated.”*
6. Leading positions		
Despite feminisation there are more men in leading positions.	6	*“More female than male employees, especially part-time. These jobs have little chances for promotion. If there is a vacancy, it is filled with a man.”*
7. Working climate		
Negative working climate because of a work surplus for male and childless doctors.	7	*“More pregnant women, more (single) mothers in part-time work, more work absences, more work for the others (men and those without children)!”*
8. Patient care		
Patients wish to be treated by a male therapist/doctor.	7	*“Male doctors and therapists are scarce in medical fields with children and adolescents. But parents specifically ask for them.”*
		*“Some parents of our young patients choose a doctor of a specific gender. Quote from a patient’s mother: “From nursery to the end of high school, there has been mainly female supervision for my son – please now not the orthodontist as well.” After saying that, they chose a male colleague.”*
11. Overall evaluation of responses	*N*^a^	
Emotionally negative responses	60	
Emotionally positive responses	8	
Emotionally neutral responses	56	
12. Perspective towards the future		
Negative perspective: I don’t believe that medicine is prepared for the change caused by feminisation or can adapt to it. I believe working conditions will be aggravated further in the future.	46	
Positive perspective: I believe there will be a change of working conditions because of feminisation. I believe working conditions will improve.	14	
Neutral perspective: Working conditions should change to adapt to the feminisation of medicine.	11	

^a^*N* Number of responses

In addition to categorizing the free-text answers, we classified the comments as “overall negative” and “overall positive” to depict the emotions participants displayed towards feminisation, which in some cases were expressed very strongly. Comments which did not indicate a clear emotion were classified as “neutral”. Less than half of the responses were rated as neutral; overwhelmingly, the most commonly expressed emotion was “negative” (Table 4).

## Discussion

Our survey with UMG applicants, students and doctors aims to give insight into how the “feminisation of medicine” is viewed within the medical community, in particular regarding its relevance and implications to the profession.

### Relevance of feminisation

To address the relevance of feminisation, participants were first asked to rate the importance of gender balance among the medical workforce (item 1) followed by an evaluation of the importance of gender for the doctor-patient-relationship from different perspectives (item 2). These questions aimed to provide insight into the understanding that current and future doctors have of their own profession, and whether this understanding in some aspects conflicts with the current trend of feminisation.

### Gender balance

The question of gender balance was answered unambiguously in all three survey groups: a balanced gender ratio among doctors was found to be “important” or “very important.” Applicants rated this more frequently as “very important” than the other survey groups. Additionally, our results showed that female participants in the student and applicant groups rated gender balance as significantly more important than men.

To our knowledge, this question has not been asked previously in the literature and therefore the relevant findings constitute a new result. Indirect explanations can be derived from non-medical studies: a US-American study investigating the job satisfaction of a random sample of 1600 employees via telephone interview concluded that staff of a gender balanced team was most satisfied [29]. A review of 7 other studies from 1987 to 1998, however, shows differing results: men were most satisfied in male-dominated teams, while interestingly women also showed a tendency to prefer a male-dominated workplace [30]. The pattern of answers in our study may be explained by increased job satisfaction when the gender ratio is balanced or more male-dominated, which appears to be especially the case for our female participants. Comparisons to our study may be limited in their usefulness as these prior studies were not specifically performed at medical workplaces; also, more recent studies on this topic are rare. Another explanation could be that ideological convictions or social desirability [31] influenced the participants to answer in favour of a balanced gender ratio, as diversity among doctors is a concept that has been described and promoted internationally [27, 32].

### Relevance of doctors’ gender for the doctor-patient relationship

Participants in our study generally rated the doctor’s gender as unimportant rather than important in establishing a bond of trust with patients.

When rated as important, students and doctors most often reasoned that gender mattered for the conducting of intimate examinations and that patients tended to open up more towards a doctor of their own gender. Literature supports these claims – many patients prefer a doctor of their own sex for intimate examinations, such as anal and genital examinations by their general practitioner [5], gynaecological [33], urological [21, 34] and endoscopic examinations [35, 36]. That patients preferentially trust doctors of the same sex follows from a review by Sandhu et al. [22], which concluded that gender-concordant doctor-patient relationships are distinguished by less tension and more ease. Psychotherapy was named as another area in which the gender of the therapist was relevant. Literature provides conflicting results as to whether patients prefer a psychotherapist of their own gender. Blow et al. [23] conclude that a patient’s preferences and experiences are distinct and a diverse pool of therapists, also in regard to gender, is essential to adapt the therapy to the individual patient.

Comments from both students and doctors also contained reports of discrimination against female doctors, with patients and colleagues rating female doctors as less competent because of their sex. This finds resonance in the literature, where men are seen as more “technically competent”; however, positive stereotypes such as social empathy are more commonly associated with female doctors. Overall, both female and male doctors seem to be similarly advantaged and harmed by stereotypes [5], but another study suggests that such stereotypes are rarely acted upon by patients in choosing a physician [37].

The most commonly given reason to rate a doctor’s gender as “unimportant” or “very unimportant” was the secondariness of gender to other attributes such as competence, character or empathy. These statements find support in literature: patients prefer a more competent doctor of either gender to a less competent doctor of the same gender [34, 38, 39]. Indirect evidence can be derived from other studies in which a substantial fraction of patients did not rate gender as an important factor in a doctor and therefore can be assumed to value other characteristics instead, even though this wasn’t directly asked of them [4, 5, 21, 33, 35, 36].

We observed a discrepancy in students’ and doctors’ assessments of the relevance of a doctor’s gender depending on the respondent’s role: when asked to judge in the role of a patient, the gender of a doctor was rated as less relevant than when in the role of a doctor, and was even underestimated given the evidence in literature. This discrepancy hints at a superordinate concept of the medical profession which influenced the participants’ answers. The Declaration of Geneva [40] dictates gender neutrality for the medical profession: the gender of the patient may not intervene with the doctor’s conduct. However, the conclusion that neither the doctor’s nor the patient’s gender may play a role in the doctor-patient relationship constitutes a fallacy. The doctor should not expect the same gender neutrality from his patient that is demanded from him; on the contrary, as part of their professionalism, doctors should be very aware of their gender and its possible impacts when dealing with patients to ensure a best possible and patient-oriented care. Considering the various educational backgrounds of the respondents of this survey, it is clear that there is a widespread deficiency in teaching gender awareness within the German medical education system. We would recommend that medical educators, faculty, staff and also national associations pull together to discuss and reflect upon gender awareness, beginning with bed-side-teaching situations, lectures or even changing curricula and learning objectives.

Our study was limited in the sample sizes from specific medical fields. Therefore, no firm conclusions could be drawn as to whether doctors of medical fields in which the doctor’s gender had been regarded in previous studies as more important (e.g. gynaecology, urology, psychiatry and general medicine) are aware of the relevance of their own gender. This would be an interesting question for further research.

### Implications of feminisation

One of the most frequent statements by our survey participants concerned the changing composition of the medical team, with an increasing number of female doctors as well as staff working part-time. This agrees with an internationally observed trend: an increasing number of female doctors (who are more prone to work part-time) results in overall reduced “hours worked” by doctors, meaning that for the same number of employed doctors, less hours have been worked. In some European countries the average hours worked per female doctor has been estimated as less than 75% of average hours worked per male doctor [41]. According to the OECD Health Working Papers, a further proportional increase in female doctors (without a corresponding increase in overall doctor numbers) would lead to an increased shortage of doctors in terms of work hours [7].

Part-time work and a feminised workforce were overall negatively connoted in our study, viewed as leading to more absences from work due to pregnancy, maternity and parental leave, while single and male doctors felt an overload of work in compensating for their female colleagues. At the same time, pregnant and female doctors felt unfairly judged.

Another aspect criticised in the open answers was a decline in diversity of the medical profession as a consequence of feminisation: the workforce was more female dominated, making it hard to accommodate special wishes of patients in regard to gender. Additionally, capacities for specialisation and research are in decline. That the feminisation of medicine is one of several important factors contributing to the so-called “crisis of academic medicine” has been described by the “International Campaign to Revitalise Academic Medicine” [8]. Women tend to place more importance in the family-friendliness of their job than in a career in academia and research.

Other respondents lamented the fact that, despite the feminisation of medicine, higher positions are still dominated by men [13]. This ambivalence between the prioritisation of family over career and the concern of a glass ceiling for women in leading positions finds resonance in the literature. On the one hand, women show different ideas and expectations towards their own profession, with a stronger wish for part-time work, a preference for working in a smaller hospital, improved work-life-balance and a reduced interest in specialising, surgery or a medical career than men [42–45]. On the other hand, there is evidence for a lack of equal opportunities for women who do strive for a career. Often, only childless women are able to pursue a medical career [46, 47], and women generally have fewer opportunities to reach higher positions than men, a phenomenon which is described as the “leaky pipeline” [48–50]. There is strong evidence that a contributing factor to the latter phenomenon is implicit gender bias and discrimination [12].

At any rate, there is agreement in politics and the literature about the need to address the “leaky pipeline”, and create opportunities for women to pursue a career besides family. A call for the adaptation of present working conditions and working-time models to the increasing number of female doctors is also made by the participants of our survey – not only to facilitate mothers to follow their medical careers, but also to avoid overloading childless and male doctors. Clearly, the increased participation of women in the medical workforce brings with it many positive implications an, as a worldwide trend, is unlikely to change in the immediate future. Nevertheless, we must also be mindful of the potentially problematic consequences of this trend, and the predominantly negative statements made regarding the trend of feminisation in this survey emphasise that current systems are poorly equipped to cope with these developments.

## Limitations

Our response rate of 26% is in line with comparable electronic surveys in Germany, but is nevertheless low. This introduces the possibility of bias amongst respondents, i.e., individuals with particularly strong opinions on this topic may have answered preferentially. As the applicant survey doubled as a pilot study that helped us generate closed questions from free-text responses, the sampling method and response rate differed from the student and doctor surveys. This limits the comparableness of the results of our survey subgroups. The makeup of the sampled students was representative of the medical student body in terms of gender ratio and stage of medical education; however, overall more female doctors participated in our study and comparatively more consultants and principal consultants than residents. Hence the representativeness of the doctor survey group to the doctors at UMG is limited.

## Conclusions

The feminisation of medicine was mostly viewed critically by the participants of this study. The respondents evaluated gender as being relevant for the medical profession and favoured a diverse workforce. However, the significance of one’s own gender in medical practice was underrated in comparison, implying a need for more awareness of the impact of a doctor’s own gender on the doctor-patient-relationship, beginning in medical school. The mainly negative comments concerning the impact of feminisation on work organisation, work satisfaction and patient care show the need for action to adapt the current medical work practices to this trend: male and childless doctors feel overloaded with work in the current model, while working mothers and mothers on work absence feel pressured and judged. With an increasing number of female students soon to be doctors, one should expect the impacts of feminisation to become only more prominent. We recommend that hospitals adapt their work system to the changing demographics of medical professionals.

Overall it should be noted that society is best served by a diverse medical profession that reflects the society in its composition in terms of ethnicity, race and gender. Our study showed that diversity in terms of gender balance is important to the members of the medical profession themselves. Further research is recommended regarding what adaptations in the workplace will improve work satisfaction and quality of patient care in the context of the increased feminisation of medicine.

## Data Availability

The datasets analyzed during the current study as available here, but in German: https://ediss.uni-goettingen.de/bitstream/handle/11858/00-1735-0000-0023-3F8C-D/Dissertation%20Dorothea%20Laurence.pdf?sequence=1 and from one of the authors: Anne Simmenroth: Simmenroth_a@ukw.de

## Abbreviations

BoK: “Basiswissengeleitete offene Kategoriefindung”, technique of categorization led through basic knowledge
OECD: Organisation for Economic Co-operation and Development
STEM: Science, technology, engineering, mathematics
UMG: Universitätsmedizin Göttingen
UQ: Unanswered question
